# Gut dysbiosis and impairment of immune system homeostasis in perinatally-exposed mice to Bisphenol A precede obese phenotype development

**DOI:** 10.1038/s41598-017-15196-w

**Published:** 2017-11-03

**Authors:** Yann Malaisé, Sandrine Menard, Christel Cartier, Eric Gaultier, Frédéric Lasserre, Corinne Lencina, Cherryl Harkat, Nancy Geoffre, Laïla Lakhal, Isabelle Castan, Maïwenn Olier, Eric Houdeau, Laurence Guzylack-Piriou

**Affiliations:** 1 Intestinal Development, Xenobiotics and ImmunoToxicology team, Toxalim (Research Centre in Food Toxicology), Université de Toulouse, INRA, ENVT, INP-Purpan, UPS, Toulouse, France; 2 Neuro-Gastroenterology and Nutrition team, Toxalim (Research Centre in Food Toxicology), Université de Toulouse, INRA, ENVT, INP-Purpan, UPS, Toulouse, France; 3 Integrative Toxicology and Metabolism team, Toxalim (Research Centre in Food Toxicology), Université de Toulouse, INRA, ENVT, INP-Purpan, UPS, Toulouse, France; 4 Adipocyte secretions, obesities and related diseases team, Institut National de la Santé et de la Recherche Médicale (INSERM), Université Paul Sabatier (UPS), Unité Mixte de Recherche (UMR) 1048, Institut des Maladies Métaboliques et Cardiovasculaires (I2MC), Toulouse, France

## Abstract

Epidemiology evidenced the Bisphenol A (BPA), a chemical found in daily consumer products, as an environmental contributor to obesity and type II diabetes (T2D) in Humans. However, the BPA-mediated effects supporting these metabolic disorders are still unknown. Knowing that obesity and T2D are associated with low-grade inflammation and gut dysbiosis, we performed a longitudinal study in mice to determine the sequential adverse effects of BPA on immune system and intestinal microbiota that could contribute to the development of metabolic disorders. We observed that perinatal exposure to BPA (50 µg/kg body weight/day) induced intestinal and systemic immune imbalances at PND45, through a decrease of Th1/Th17 cell frequencies in the *lamina propria* concomitant to an increase of splenic Th1/Th17 immune responses. These early effects are associated with an altered glucose sensitivity, a defect of IgA secretion into faeces and a fall of faecal bifidobacteria relative to control mice. Such BPA-mediated events precede infiltration of pro-inflammatory M1 macrophages in gonadal white adipose tissue appearing with ageing, together with a decreased insulin sensitivity and an increased weight gain. Our findings provide a better understanding of the sequential events provoked by perinatal exposure to BPA that could support metabolic disorder development in later life.

## Introduction

Obesity is an increasing health concern worldwide and poses a major risk in the progress of type II diabetes (T2D), cardiovascular disease and hypertension^1^. Risk factors such as bad dietary habits, lack of physical activity or genetic factors, and environmental chemicals with endocrine-disrupting activity may promote adipogenesis and weight gain^2,3^. Among these products, bisphenol A (BPA) has been reported as a potential determinant of obesity epidemic^4,5^. BPA is omnipresent in our daily life due to its wide use in manufacturing polycarbonate plastics, epoxy resins as well as in thermal printing papers, making BPA exposure ubiquitous for humans due to these multiple sources and daily contact. Accordingly, BPA is detectable in the urine but also in the placenta, umbilical blood, amniotic fluid and breast milk in humans, at relevant concentrations for health concern for the foetus, then for breastfed child^6,7^. Indeed, animal studies emphasized that perinatal exposure to low doses of BPA increases the risk of metabolic disorders in offspring, making this period of development a critical window of exposure to this chemical^8,9^. In Humans, high BPA levels have been associated with obesity and T2D, and the economic impacts of obesity due to BPA have been recently estimated to represent 42 000 new cases and € 1.54 billion loss by year in Europe^5,10–13^.

How perinatal exposure to BPA contributes to metabolic disorders in adulthood is still unclear. One of the major drivers in obesity and diabetes is inflammation^14^. In obese mice and humans, white adipose tissue (WAT) presents infiltrating M1 macrophages that secrete pro-inflammatory cytokines such as TNF-α and IL-6, characterizing chronic low-grade inflammation in parallel to adipogenesis and weight gain^15,16^. Moreover, the Th1/Th2 cell ratio is increased in obesity, proved by increased IFN-γ production in obese mice^17^. Perinatal exposure to BPA in rats has been shown to deeply affect homeostasis of the gut immune system^18,19^, and has been also regarded as a risk factor of developing pro-inflammatory conditions in adult life^20^. Altogether, these observations underline a possible link between perinatal exposure to BPA, inflammation and the development of obesity later in life.

On the other hand, obesity and T2D are also found associated with intestinal dysbiosis, suggesting that the microbiota is crucial for the metabolic development of the host. Indeed, conventionalization of germ-free mice increases about 57% the total body fat and increases insulin resistance^21^. Furthermore, *ob/ob* mice and obese humans displayed an increased ratio *Firmicutes*-to-*Bacteroidetes* compared to lean individuals, suggesting an obese-leading gut dysbiosis^22^. This is consistent with a recent case report showing obesity development in a woman after faecal microbial transplantation from a pre-obese subject^23^. Faecal transfer from obese mice to germ-free mice also increased adiposity of recipients compared to faecal transfer from lean mice to germ-free mice^22^. Regarding BPA exposure, a role in dysbiosis-induced obesity could exist since perinatal exposure not only affects the immune system but also the gut microbiome in adult life^24,25^. The microbiota plays also a major role in inflammation because of its tight relationship with immune system development and maturation^26^. However, reports on BPA effects analysed these parameters separately or together through transversal studies in adults, i.e., without chronological consideration. It should be hypothesized that a possible multi-effect of BPA on immune system development, gut microbiota and obesity may be sequential with immune system dysregulation and/or intestinal dysbiosis preceding the BPA-related obesogenic effects. To this purpose, we performed a longitudinal study in mice aimed at investigating the immunologic, microbial and metabolic parameters from early adulthood ((Postnatal day (PND) 45) until ageing (PND170) after perinatal exposure to BPA.

## Results

### Perinatal exposure to BPA contributes to obese phenotype and metabolic disorders at adulthood

**Figure 1:**
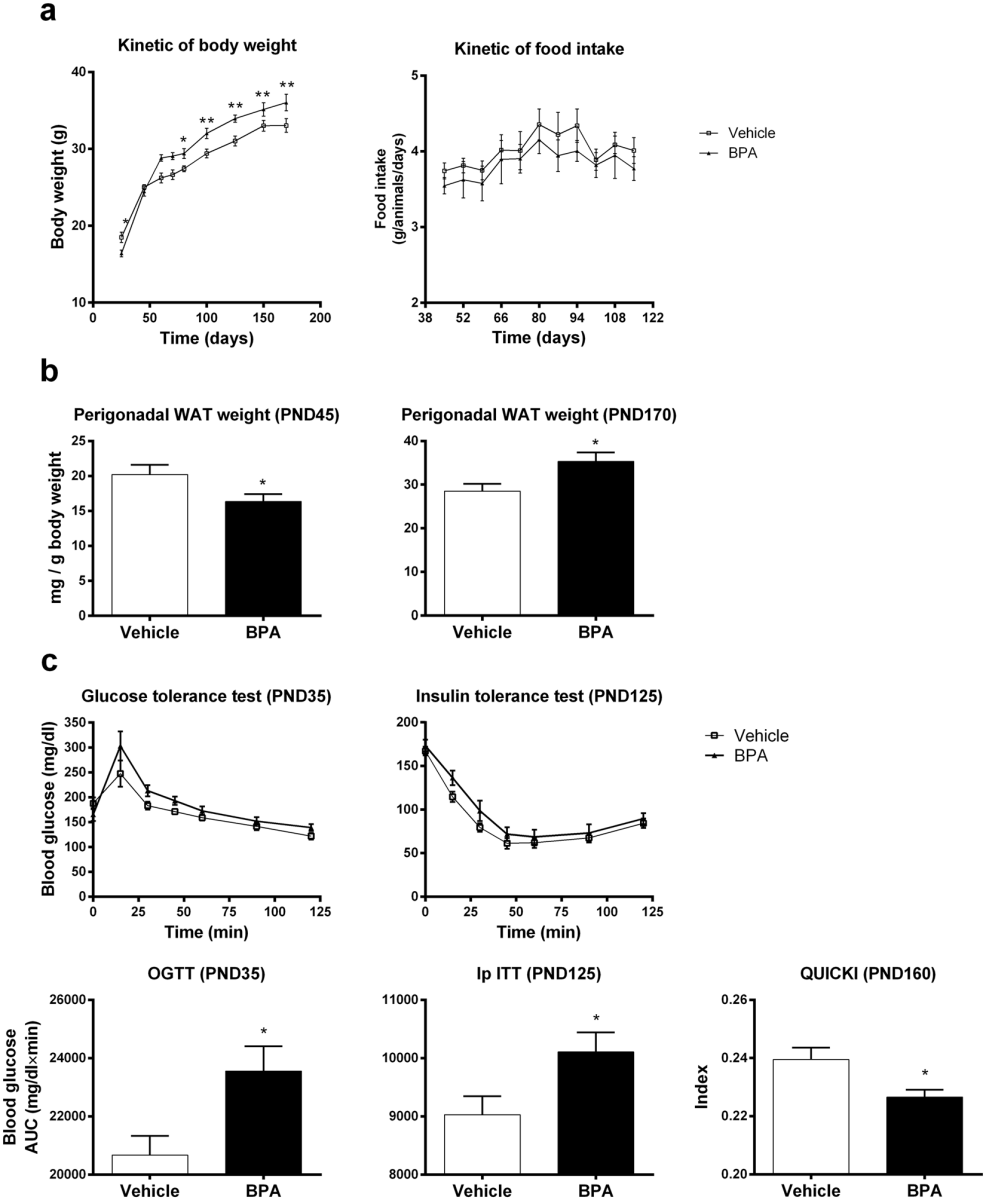
Perinatal exposure to BPA provokes metabolic disorders in offspring male mice. (**a**) Body weight of BPA and vehicle mice was measured from PND21 to PND170. (**b**) Perigonadal WAT weight of BPA and vehicle offspring was determined at PND45 and PND170. (**c**) Glucose and insulin tolerance tests were performed at PND35 and 125 respectively. The Quantitative Insulin Sensitivity Check Index (QUICKI) was calculated at PND160. Data represent the mean ± s.e.m. (n = 10–16). Data were representative from two batches of experiments. *P < 0.05, **P < 0.01, vs. non-exposed group using a Mann-Whitney’s post-hoc analysis.

We studied first the effect of perinatal exposure to BPA on body weight under normal diet (Fig. 1a). We noted that at early life (PND25), mice perinatally exposed to BPA (BPA offspring) were significantly leaner than vehicle offspring mice, associated with a decrease of gWAT at PND45 in BPA offspring. However, from PND70 until PND170, BPA offspring exhibited a significant rise in weight gain compared to vehicle offspring. Although perinatal exposure to BPA increased the body weight of mice, it did not change the food intake (Fig. 1a). The weight gain in BPA offspring was associated with an increase of gWAT weight at PND170, which was not observed at PND45 (Fig. 1b).

**Figure 2:**
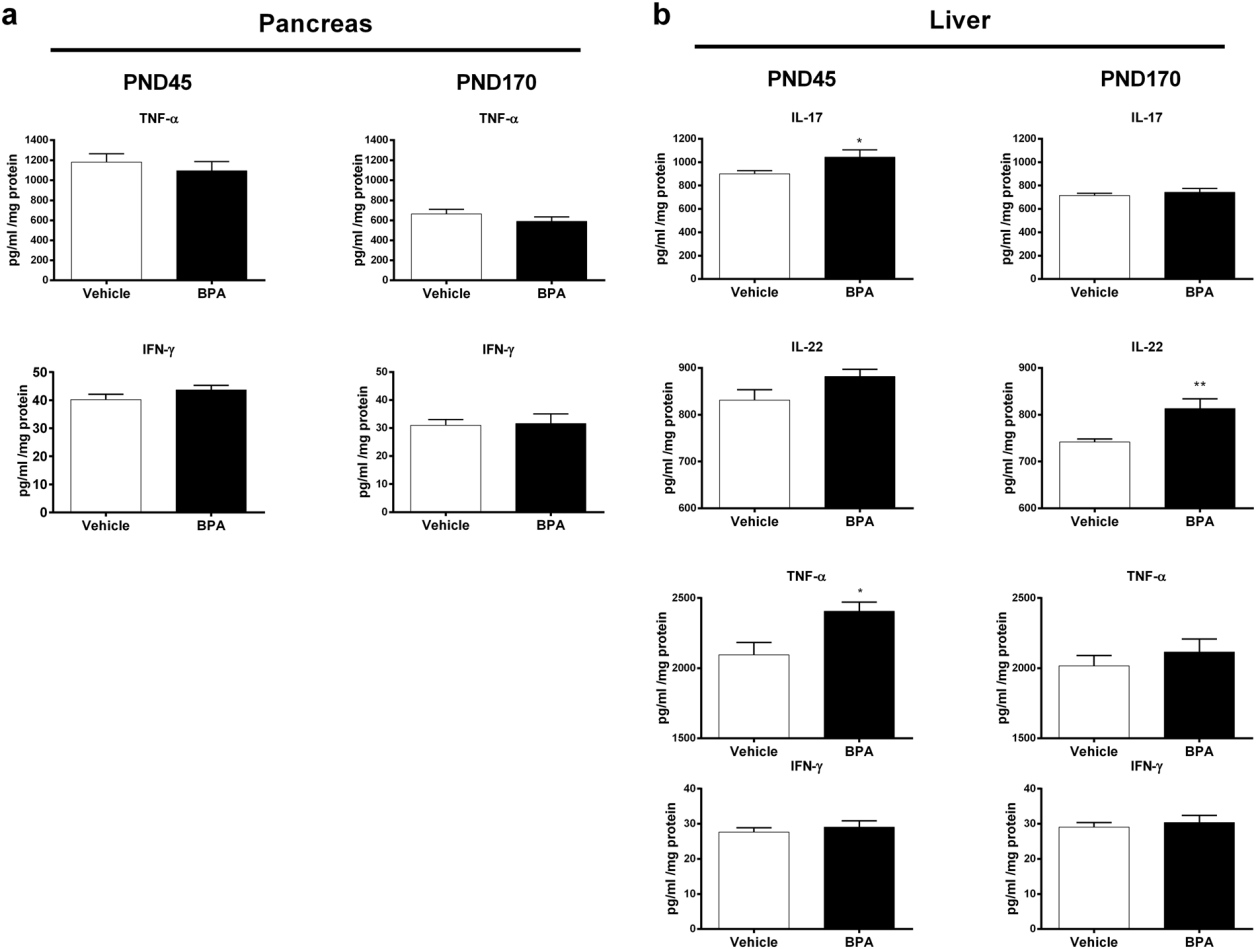
The metabolic disorders due to BPA is associated with liver but not with pancreas inflammation. At PND45 and PND170, cytokine levels were determined in pancreas (TNF-α and IFN-γ) (**a**) and in liver (IL-17, IL-22, TNF-α and IFN-γ) (**b**) of BPA and vehicle exposed offspring. Data represent the mean ± s.e.m. (n = 10–16). Data were representative from two batches of experiments. *P < 0.05, **P < 0.01, vs. non-exposed group using a Mann-Whitney’s post-hoc analysis.

At PND35, BPA offspring developed first significant glucose intolerance (Fig. 1c). The disturbance in glucose tolerance was followed by a decrease of the insulin sensitivity at PND125. We also observed a decrease of the Quantitative Insulin Sensitivity Check Index (QUICKI) in these animals at PND160. However, no significant differences on inflammatory cytokines TNF-α and IFN-γ level were detected in the pancreas between the two groups regardless of age of mice (Fig. 2a). We then determined the level of IL-17, IL-22, TNF-α and IFN-γ in liver. BPA perinatal exposure induced at PND45 hepatic inflammation in male offspring compared to vehicle group characterized by an increase of IL-17 and TNF-α levels, which returns to baseline at PND170 (Fig. 2b). A trend to increased IL-22 levels was observed at PND45 compared to vehicle offspring, and became significant in later life at PND170. IFN-γ level did not change whatever the age of mice.

**Figure 3:**
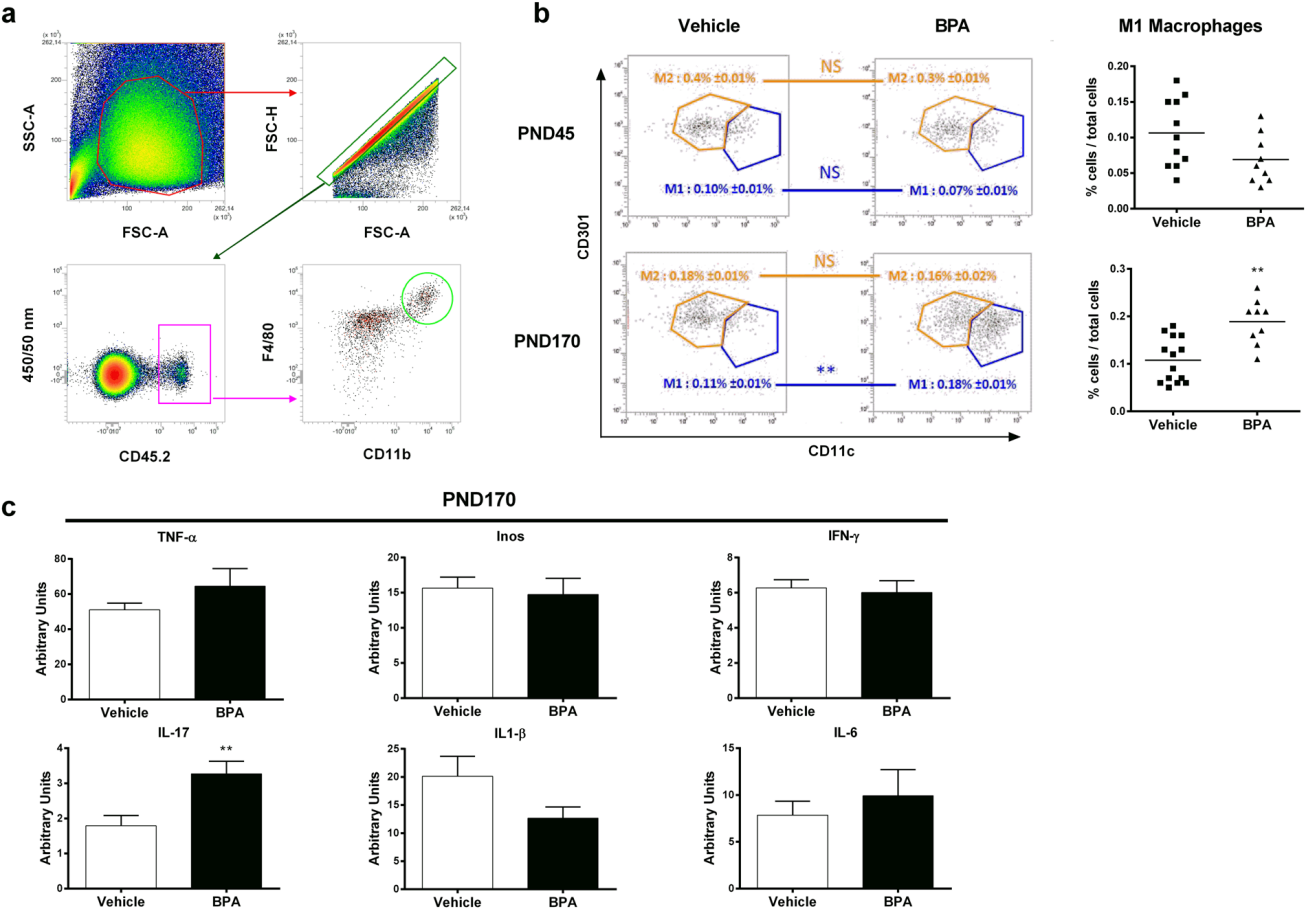
Perinatal exposure to BPA induces inflammated-M1 deviation in gonadal WAT associated with IL-17 inflammation. (**a**) Gating strategies for perigonadal WAT macrophages (**b**) M1 and M2 macrophages were phenotypically characterized from CD45.2^+^ F4/80^+^ CD11b^+^ cells in perigonadal WAT of BPA and vehicle-exposed offspring at PND45 and PND170. (**c**) TNF-α, Inos, IFN-γ, IL-17, IL1-β and IL-8 expression levels were determined in perigonadal WAT by RT-PCR at PND170. Data represent the mean ± s.e.m. (n = 10–16). Data were representative from two batches of experiments. **P < 0.01, vs. non-exposed group using a Mann-Whitney’s post-hoc analysis.

Therefore, we isolated macrophages from gWAT and discriminated M1 and M2 macrophages subsets (Fig. 3a,b). We found a significant increase of the percentage of inflammatory M1 macrophages in gWAT of BPA offspring at PND170, but not at PND45, relative to the control vehicle group, without changes in anti-inflammatory M2 macrophages frequency. Those results were validated by isotype control assessment (Supplementary Figure S1). Only the IL-17 expression was increased in gWAT of BPA offspring compared to the vehicle group (Fig. 3c), whereas all others tested genes were unaffected.

### BPA perinatal exposure affect local and systemic immune response

**Figure 4:**
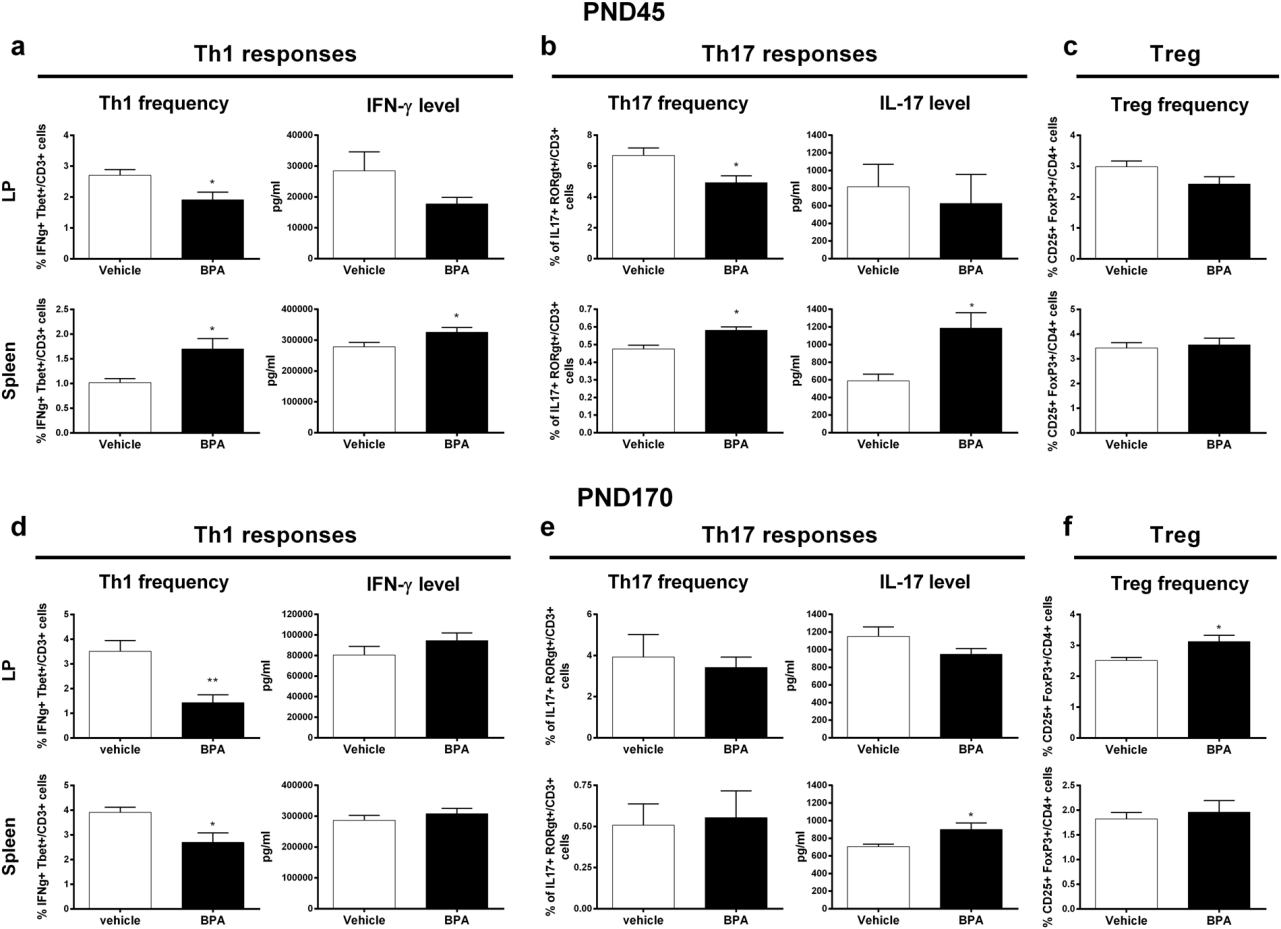
Perinatal BPA exposure disturbs the immune homeostasis in LP and spleen. Proportion of CD3^+^ Tbet^+^ IFN-γ^+^ Th1 cells, CD3^+^ RORγt^+^ IL-17^+^ Th17 cells and CD4^+^ CD25^+^ FoxP3^+^ Treg cells in LP and spleen of BPA and vehicle exposed offspring at PND45 (**a**–**c**) and PND170 (**d**–**f**). IFN-γ and IL-17 levels measured at PND45 and PND170 in supernatant of isolated LP cells and splenocytes after 3 days of *in vitro* anti-CD3/CD28 stimulation. Data represent the mean ± s.e.m. (n = 8–16). Data were representative from two batches of experiments. *P < 0.05, **P < 0.01, vs. non-exposed group using a Mann-Whitney’s post-hoc analysis.

Perinatal BPA induced a significant drop of Th1 frequency in the *lamina propria* (LP) at PND45, associated to a trend to a decreased IFN-γ secretion after anti-CD3/CD28 *in vitro* re-stimulation compared to vehicle group (Fig. 4a). Moreover, the Th17 subsets significantly decreased in the LP of BPA offspring with no change in IL-17 secretion and Treg frequency, compared to vehicle group (Fig. 4b,c). On the other hand, we noticed a significant increase of Th1 and Th17 subsets in association with IFN-γ and IL-17 secretion after *in vitro* re-stimulation of splenocytes from BPA offspring compared to vehicle group (Fig. 4a,b). No change was observed in Treg frequency in this compartment (Fig. 4c).

At PND170, in the LP of BPA offspring, we observed a significant decrease of Th1 subset frequency associated with an increase of Treg and no change in Th17 cell frequency or IL-17/IFN-γ productions in response to CD3/CD28 (Fig. 4d,e,f). Furthermore, we found a significant decrease of Th1 frequency in splenocytes from BPA offspring, without association with IFN-γ level (Fig. 4d). Treg and Th17 frequencies did not change in BPA offspring while the IL-17 level in systemic compartment was significantly higher at PND170 compared to vehicle group (Fig. 4e,f).

### Perinatal exposure to BPA impairs local IgA production and antimicrobial activity in parallel to faecal dysbiosis

**Figure 5:**
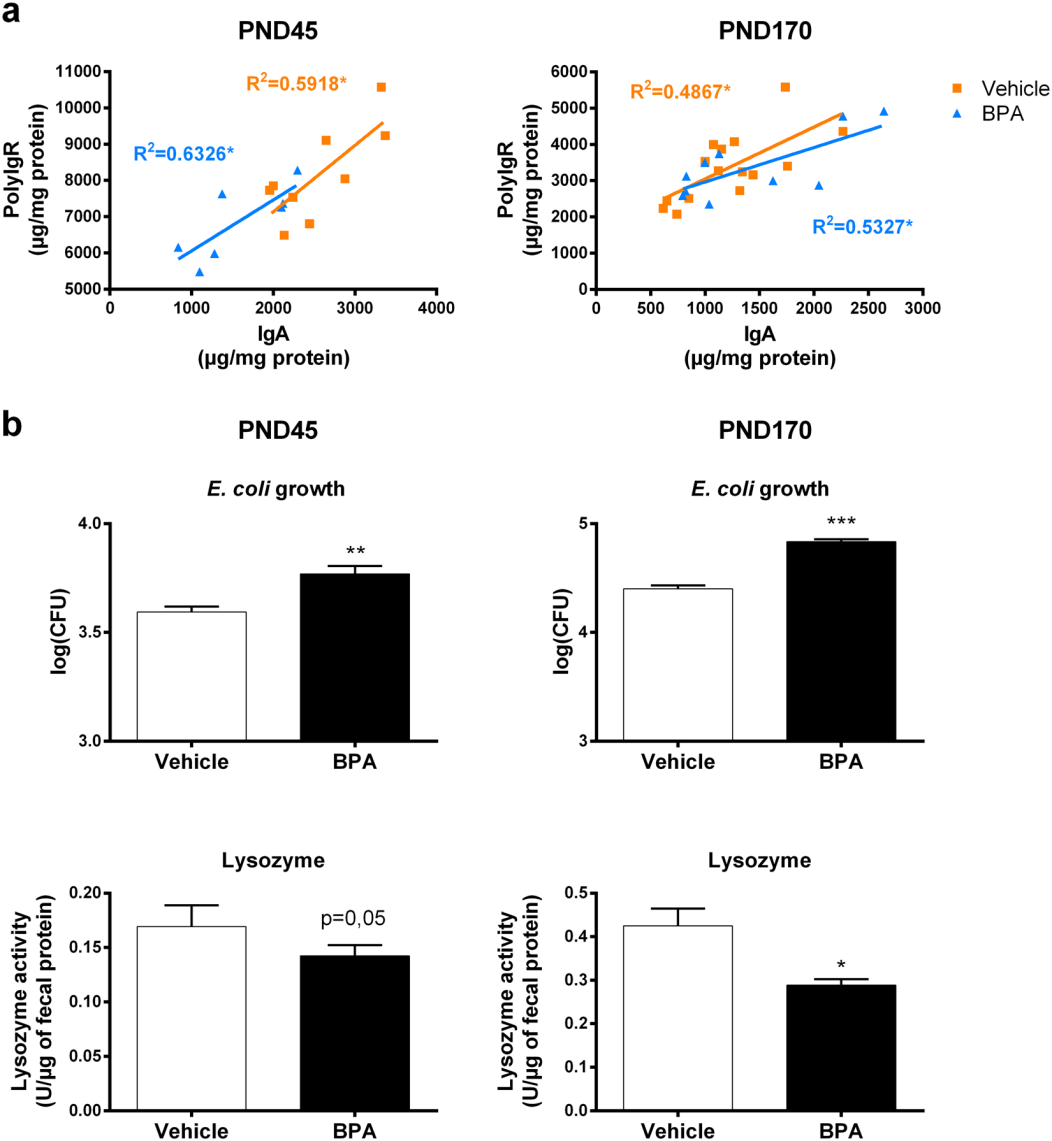
Perinatal exposure to BPA impairs local IgA production and antimicrobial activity leading to dysbiosis in faeces. (**a**) Faecal IgA and polyIgR levels were assessed and a linear regression has been made to correlate these two parameters in BPA and vehicle exposed offspring at PND45 and PND170. (**b**) Impact of 1 mg of faecal supernatant protein on commensal *E. coli* growth and lysozyme activity in faecal supernatant from BPA and vehicle exposed offspring at PND45 and PND170. Data represent the mean ± s.e.m. (n = 8–16). Data were representative from two batches of experiments. *P < 0.05, **P < 0.01, ***P < 0.001 vs. non-exposed group using a Mann-Whitney’s post-hoc analysis.

We then investigated the impact of perinatal exposure to BPA on intestinal barrier defences and its possible impact on the microbiota. We investigate first the IgA secretion and the analysis of pIgR level in faeces. pIgR performs the transfer of IgA to the lumen from gut mucosa^27^. We observed a decrease in IgA production at PND45 in faeces of BPA offspring compared to vehicle group. This decrease was associated with a default of pIgR production, which was found restored at PND170 (Fig. 5a). Since IgA are important for the gut barrier integrity and the homeostasis between host and gut microbiota, we then determined the faecal antimicrobial activity against commensal *Escherichia coli* (*E. coli)* (Fig. 5b). Faecal supernatant of BPA mice allowed more *E. coli* colony formation than faecal supernatant of vehicle mice at PND45 and PND170, meaning that faecal antimicrobial activity against commensal *E. coli* is decreased in these animals. We also observed a significant decrease in the faecal lysozyme activity against peptidoglycan in BPA offspring at PND170, and a strong trend to the decrease at PND45, compared to vehicle group. Those effects occurred without any change in intestinal permeability (Supplementary Figure S2).

**Figure 6:**
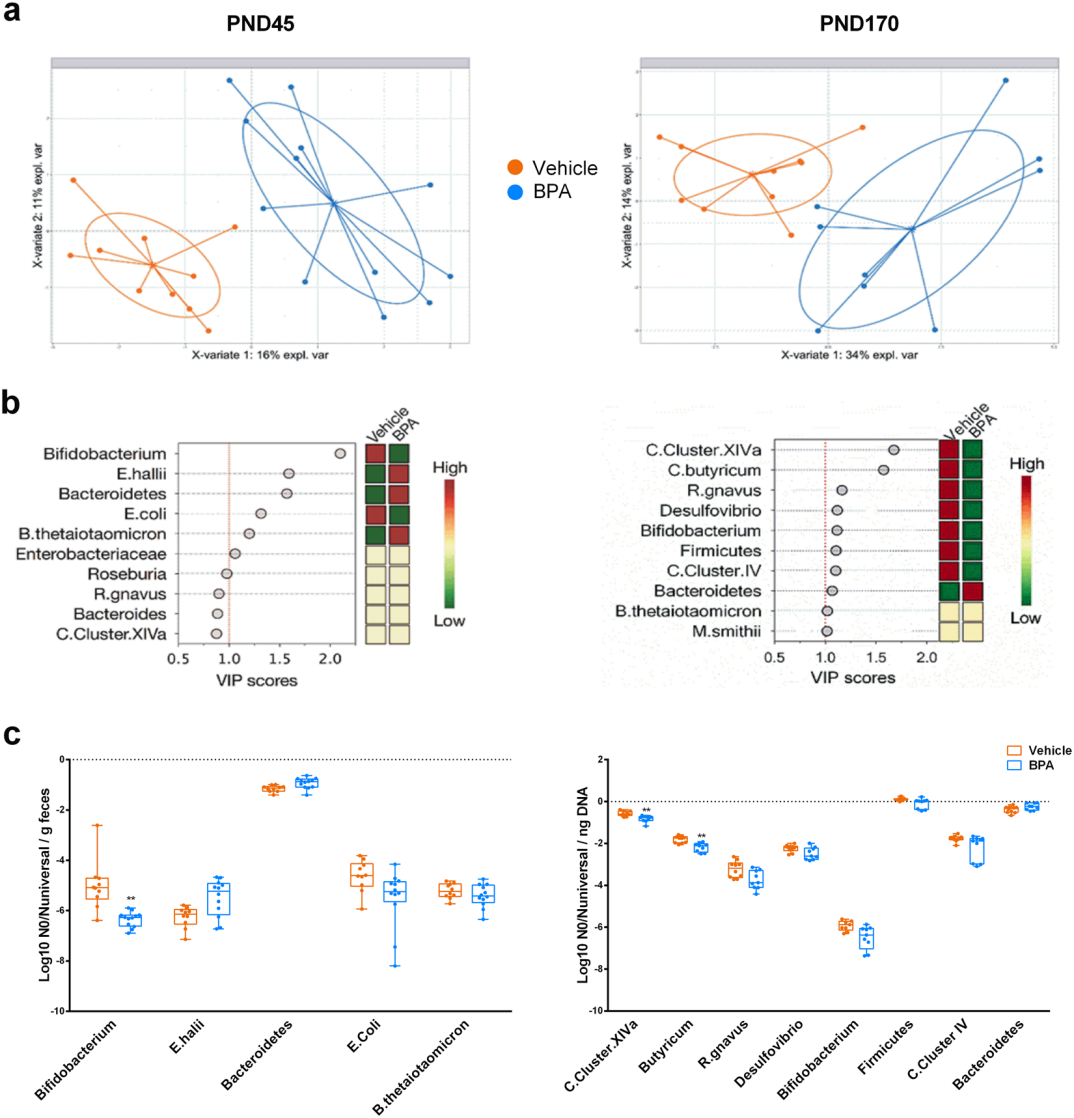
Faecal microbial alterations in response to perinatal exposure of BPA in mice offspring at PND45 and PND170. (**a**) PLS-DA score plots of the relative quantitative abundances (Log10 No) of twenty-two microbial taxa in faeces of BPA and vehicle offspring. (**b**) VIP plot representing the ten most discriminating features (microbial taxa) identified by PLS-DA build models at PND45 and PND170 (n = 9–10). (**c**) Relative abundances per ng DNA of microbial taxa with a VIP > 1 by real-time PCR. **P < 0.01, vs. non-exposed group using a Mann-Whitney’s post-hoc analysis and adjustment using Benjamini-Hochberg’s method.

We further study whether the BPA-mediated changes in antimicrobial activity could affect the gut microbiota. Score plot shows a clear separation between the microbial profiles of mice according to perinatal intervention both at PND45 and PND170 (Fig. 6a). Among the targeted microbial communities, *Bifidobacterium spp*. was the most important contributor to the observed differences at PND45 (Fig. 6b). We reported a significant decrease of bifidobacteria in BPA offspring at PND45 compared to vehicle group (Fig. 6c). We further observed the decline in bifidobacteria at PND170 in BPA offspring associated to a significant drop in the phylum of Firmicutes, among which more precisely the relative abundances of *Clostridium butyricum* and *Clostridium Cluster XIVa* were significantly affected (Fig. 6b,c).

## Discussion

The obesogenic effect of the chemical BPA for humans is still under debate while animal studies commonly reported correlation between perinatal exposure to BPA and metabolic disorders throughout life. However, the mechanisms by which BPA exposure leads to diabetes and obesity are not understood yet. Recent data suggested that the immune system, by promoting inflammation, is closely linked to metabolic diseases and obesity developments^14–16^. These observations of a close relationship between immune and metabolic status lead to an emerging field of research named “immunometabolism”^28^. It became clear that low-grade inflammation is a key feature of obesity and T2D by the fact that the excess of pro-inflammatory cytokines impairs cellular insulin signalling^29–34^. Both innate and adaptive immune cells contribute to metabolic disorders. In the gut, CD4^+^ T cells contribute to immunity by differentiating into various subsets, notably inflammatory (Th17/Th1) and regulatory cells (Treg), Th17 cells being the most abundant CD4^+^ T cells in mucosal tissues^35–37^. They secrete isoforms of IL-17 and/or IL-22, which confer protection against fungi and pathogenic bacteria. BPA’s role in the occurrence of metabolic disorders associated with inflammation remains unclear while a consensus exists that the perinatal period is a critical window for BPA exposure imprinting immune system development and function for life^8,9,18–20^. The gut microbiota plays also a crucial role in the development of obesity and diabetes, and its relationship with the immune system makes it a great point of interest. Moreover, it is admitted that male mice are more affected by diabetes and obesity than in females^38,39^. In this report, we performed a longitudinal study of male mice perinatally-exposed to BPA to better understand the chronological order of appearance of immunologic, microbial and metabolic disturbances at adulthood (PND45) and at ageing (PND170).

Firstly, we confirmed the obesogenic impact of *in utero* and early life exposure to BPA at a dose of 50 µg/kg body weight (BW)/day, which is 100 times below the current no observed adverse effect level (NOAEL, 5 mg/kg BW/day), in male mice. In our study, young mice perinatally exposed to BPA were leaner and exhibited less perigonadal WAT than vehicle offspring, which have been already reported in other studies^40,41^. However BPA mice recovered rapidly to a normal weight that drifted into an increased body and gWAT weight compared to vehicle offspring. Intolerance to glucose and decreased insulin sensitivity was observed in young and elder mice perinatally exposed to BPA. Indeed, the observed decrease QUICKI in BPA offspring at PND160 reflects their greater insulin resistance^42^. Similar metabolic disorders have been described in BPA-exposed animals^8^. We could not associate this obese phenotype with pancreatic inflammation whatever the age of mice. Nevertheless, a recent study showed that BPA exposure is associated with an increase in pancreatic β-cell proliferation in early life but not at PND120; the authors explain that an excess of insulin signalling during early life may contribute to impaired glucose tolerance during adulthood^40^. Interestingly, we provide evidence that perinatal exposure to BPA leads to metabolic disorders and obese phenotype in association with gWAT inflammation *via* M1 macrophages infiltration in ageing offspring, as observed in obese humans^15,16^. Similar results were obtained in *ex vivo* culture of bone marrow differentiated macrophages from C57BL/6 mice and cultured in presence of BPA^43^. In our study, the M1 macrophages infiltration is associated with IL-17 overexpression in gWAT. This finding is concordant with studies showing tight correlation between IL-17 inflammation in WAT and obesity^44,45^. All these results confirm the obesogenic effect of perinatal exposure to BPA in offspring mice, with close characteristics to human obese phenotype, hence validate our *in vivo* model to study the chronological development of immune and gut microbiota imbalance associated with this phenotype.

In adult offspring (PND45), we showed that perinatal exposure to BPA provokes important local and systemic immune homeostasis disturbances associated with metabolic disorders, which precede the obese phenotype herein described in ageing offspring. In fact, adult BPA-exposed offspring mice displayed a lean phenotype without M1 infiltration in gWAT. However, this phenotype is associated with glucose intolerance and a systemic inflammation characterized by an increase of splenic Th1 and Th17 responses. These results are concordant with Luo *et al*. study that showed similar Th1/Th17 immune deviation in spleen of male and female mouse offspring perinatally exposed to BPA^46^. Moreover, we showed that BPA perinatal exposure leads to a loss of Th1/Th17 cell subsets from LP. Surprisingly, less pronounced immune homeostasis disturbances in intestinal and systemic compartments were observed in ageing BPA offspring. However, ageing BPA offspring conserved their increased production of IL-17 in spleen. Interestingly, recent publications showed similar features in mice fed with high-fat diet (HFD)^47,48^. Of note, Garidou *et al*. reported the HFD-derived ileum microbiota responsible for a decrease in Th17 cells of the LP. These authors concluded that the microbiota of the ileum can regulate Th17 cell homeostasis in the small intestine, hence determining the outcome of metabolic disease^47^.

In our study, the BPA-evoked decrease of Th17 and Th1 responses in the small intestine is associated with intestinal dysbiosis at adulthood. Indeed, we demonstrated that perinatal exposure to BPA altered the gut microbiota development in offspring mice, characterized notably by a decrease of bifidobacteria. A causal link between gut dysbiosis and metabolic diseases has been well established in rodents as well as in humans through microbiota transfer experiments^22,23^. Of importance, some strains of the genus *Bifidobacterium* has been previously described to display anti-inflammatory properties^49^. This suggests that the BPA-induced decrease of this strain in offspring could contribute to the systemic inflammation observed from PND45. Consistent with this idea, specific strains of *bifidobacteria species* are used as promising anti-obesogenic probiotics and are reported to be decreased in mice that exhibited an obese phenotype after four weeks of HFD^50–52^. Furthermore, we observed an “obese like” gut dysbiosis characterized by a loss of *C. butyricum* and *C. Cluster XIVa* among Clostridia in addition to a decrease of *Bifidobacterium spp*., in ageing BPA offspring (i.e., PND170). Our results are concordant with Lai *et al*. study using direct BPA exposure protocol, and with the fact that T2D patients are also known to display low amounts of *Clostridia* compared to healthy individuals^24^. Moreover, in their study, Lai *et al*. described a similar gut dysbiosis between mice directly exposed to BPA and high-fat or high-sucrose diet mice^24^. Considering the results of our present study, we can hypothesize that microbial changes mainly characterized by the fall of bifidobacteria or/and the disturbance of intestinal immune homeostasis in young adult (i.e., PND45) is/are responsible of altered microbial pattern observed in ageing mice (i.e., PND170). The altered gut microbiota following perinatal BPA treatment could also result in a loss of intestinal defences. Indeed, we found a decrease of IgA production and of anti-microbial activity, especially of lysozyme activity, in faeces of adult mice. These animals also present less pIgR (i.e. the receptor that performs the transfer of IgA to the lumen from gut mucosa), which could explain the decrease of IgA levels in faeces. Therefore, the loss of IgA could lead to the dysbiosis observed in these individuals. Further investigations will be necessary to determine how BPA affects pIgR expression and gut defences.

In young adult (PND45) offspring perinatally exposed to BPA, a systemic inflammation is found associated with an increase of IL-17 and TNF-α level in the liver. This effect is not observed with ageing at PND170 in contrast to IL-22 liver content that significantly rose in aged mice only after BPA exposure. Interestingly, a similar cytokine profile has been reported in Non-Alcoholic-Steato Hepatitis (NASH) (i.e., a disease associated with obesity) with IL-17-driven liver inflammation^53^. In NASH, an increase of Th17 cells during the first step of disease (i.e., when the liver begins to be damaged) precedes infiltration of “hepatoprotective” IL-22 secreting cells, and a second expansion of Th17 cells^54–56^. In our study, one may hypothesized that IL-17 inflammation in the liver of BPA mice at PND45 may induce early tissue damages that lead to secondary IL-22 inflammation with ageing. Further investigations are needed to confirm this sequence of events.

To conclude, this study showed that mice exposed to BPA during the perinatal life displayed immune homeostasis disturbances in association with gut dysbiosis in early adulthood and altered glucose tolerance, and that these disturbances occurred before the development of obese phenotype with ageing. These findings permit a better understanding of the chronological events associated with the development of obese phenotype due to BPA perinatal exposure. These data open new fields of studies to test whether early disturbances in immune system and gut microbiota related to perinatal exposure to BPA may be regarded as prognostic factors of metabolic disorder development.

## Methods

### Animals and treatments

All animals were kept at a constant temperature of 20 ± 1 °C, maintained on a 12-h light-dark cycle (lights on at 7:00 AM) and humidity-controlled facility, and housed in BPA-free polypropylene cages on corn pop bedding to avoid any cross-contamination by BPA^18^. Food (Harlan Global Rodent Diet 2018; Harlan Bioproducts, Gannat, France) and tap water (contained in polypropylene bottles) were available *ad libitum*.

Eight-week-old female and males C3H/HeN mice were purchased from Janvier (Saint Berthevin, France). After a week of acclimation, females were time-mated to eight-week-old males.

Gravid Mothers (n = 8–12 per group) from two independent series of experiments were daily treated orally from gestation day 15 to weaning of pups (PND21) with 50 µg/kg BW/d of BPA (Sigma-Aldrich, Saint-Quentin Fallavier, France) or the vehicle alone (0,1% ethanol in corn oil) as control group. In this study, this period of exposure has been defined as perinatal period. After weaning, males from different mothers but with the same treatment were cohoused 5–6 per cage to limit litter effects and body weight and food intake were weekly monitored for the duration of the study (i.e., until PND170). We will refer to these groups as BPA and vehicle offspring, respectively.

### Ethics approval

All experiments were approved by the relevant Animal Care and Use Committee (CEEA-86, Ministry of Research and Higher Education, France); notification TOXCOM/0114/LG EH, and conducted in accordance with the European directive 2010/63/UE.

### Glucose and insulin tolerance tests

Oral glucose tolerance test (OGTT) and intra-peritoneal insulin test (IpITT) were performed for the vehicle and BPA group (n = 10–16 males) at PND35 and PND125 as previously described^57^. The Quantitative Insulin Sensitivity Check Index (QUICKI) was calculated at PND160 as previously described^42^. Briefly, fasting plasma insulin (I(0)) and glucose (G(0)) were measured for the two groups of mice. The QUICKI was then calculated using the equation QUICKI = 1/[log(I(0) + log (G(0))].

### Anti-microbial activity against commensal *Escherichia coli* (*E. coli*)

The anti-microbial activity was measured as previously described^58,59^. Briefly, PND45 and 170 faeces were weighed and ground in a volume of 20% ethanol equal to 10 fold the faeces weight for a better solvability of antimicrobial peptides. The mixture was centrifuged 10 min at 1600 × g at 4 °C and the supernatant was assessed for anti-microbial activity. Live *E. coli* were isolated from faeces of naïve healthy C3H/HeN mice by culture on selective ChromID coli plates (Biomérieux, Marcy L’étoile, France). Grown colonies belonged to B1 phylogenetic group. Further characterization of one representative isolate revealed to belong to the O18 serogroup and none of checked virulence genes was harbored (*lt, sTa, sTb, stx1, stx2, eae, hly, cnf, Afa, cdt, pks*). 10^5^ colony forming units (CFU) of this commensal *E. coli* grown to mid-logarithmic phase were co-incubated with 1 mg of faecal material. The number of viable bacteria remaining after 2 h of incubation at 37 °C was analysed by plating serial dilutions on LB agar plates (BD, Le Pont De Claix, France). Results were representative from the antimicrobial activity only against the commensal *E. coli*, since any bacterial growth was observed on LB agar when prepared faecal supernatants were plated alone (i.e. without commensal *E. coli*).

### Lysozyme activity

Faecal proteins were mechanically extracted after rehydration of frozen faeces in 500 µl of PBS (10 mM; pH 7.2). After 10 minutes of centrifugation at 1600 g, supernatants were sterilized with a 0.22 µm filter and frozen (−80 °C). Faecal protein concentration was measured using BCA protein Assay kit, Uptima (Interchim, Montluçon, France). Activity of lysozyme against the peptidoglycan was determined using the EnzChek® Lysozyme Assay Kit (Molecular probes, life technology, St Aubin, France).

### Faecal microbial communities assessment

Faeces of BPA and vehicle groups were collected and weighted at PND45 and 170 for microbial characterization.

In order to obtain a microbial pattern associated to BPA exposure, an adapted protocol of the GUt low-density array (GULDA) approach developed by Bergström *et al*. has been performed^60^. PCR conditions, primer sequences and concentrations were used as previously described^61^. The normalized No-values were reported per ng of DNA, log10-transformed and processed by MixOmics package (6.1.1 version) with RStudio software (1.0.44 version) to build a partial least-squares discriminant analysis (PLS-DA)^62^. Missing normalized No-values were reconstituted using the NIPALS algorithm and M-fold cross-validation was used to select the optimal number of latent variables for PLS-DA models with minimal error rate. Variable Importance in Projection (VIP, weighted sum of squares of the PLS loadings) scores were estimated and allowed to classify the microbial amplicon groups according to their explanatory power of class label (Y = mice treatments)^63^.

### Necropsy

Animals were weighed and sacrificed at PND45 or PND170. Spleen, *lamina propria* (LP) and gonadal WAT (gWAT) were stored at 4 °C in 0.9% saline until the end of sampling and quickly used for flow cytometry or cytokine analysis. Pancreas and liver were frozen in liquid nitrogen and stored at −80 °C until cytokine assays.

### Flow cytometry

Splenic cells were isolated through a 70 µm nylon mesh in PBS and 1% KnockOut Serum Replacement (Gibco, Lifetechnologies, Paisley, UK). Cells were pelleted by centrifugation at 500 × g for 10 min at 4 °C. Gonadal WATs were digested by type II collagenase (Sigma-Aldrich) in 1% BSA HBSS at 37 °C for 30 min. The mixture was filtered through a 100 µm mesh and cells were pelleted by centrifugation at 500 × g for 10 min at 4 °C. Small intestines were washed in cold PBS, cut into 0.5 cm pieces, incubated four times in 30 ml of PBS 3 mM EDTA (Sigma-Aldrich) for 10 min at 37 °C and digested in 20 ml of RPMI 1640 added with 20% FCS and 100 U/mL of collagenase (Sigma-Aldrich) for 40 min at 37 °C. LP cells were purified on a 40–80% Percoll gradient run for 15 min at 1800 × g at RT. All cells have been checked for viability by trypan blue staining. For Th17 and Th1 intracellular staining, cells were stimulated with a cocktail of phorbol 12-myristate 13-acetate (15 nM, Sigma) and ionomycin (1 μg/ml, Sigma) for 5 h at 37 °C. Brefeldin A (10 μg/ml; Sigma) was added for 2 h after PMA-ionomycin stimulation. Cells were then stained with antibodies to the following markers from BD (BD Biosciences, San Jose, CA, USA): anti-CD4 (GK1.5), anti-CD25 (PC61), anti-CD3 (145-2C11), anti-IFN-γ (XMG1.2), anti-Tbet (O4-46), anti-CD3 (145-2C11), anti-IL-17 (TC11-18H10), anti-RORγt (Q31-378), anti-CD11b (M1/70), anti-CD45.2 (104) and anti-CD11c (HL3); and from Ebioscience (Ebioscience, Lutterworth, UK): anti-CD301 (11A10-B7), anti-F4/80 (BM8), anti-FoxP3 (FJK-16s). Specificity of each labelling was checked using specific isotype controls listed in Supplementary Table 1. The intracellular staining was realized by using a Cytofix/Cytoperm Kit Plus (BD) according to the manufacturer’s instructions. Flow cytometry data were collected on MACSQuant Analyzer 10 (Miltenyi Biotec, San Diego, CA, USA). Data were analysed using VenturiOne (AppliedCytometry, Sacramento, CA, USA) software. Lymphocytes or macrophages were selected by side scatter (SSC) and forward scatter (FSC) gating to avoid debris and dead cells. Forward scatter area (FSC-A) and height (FSC-H) gating was then used to avoid cell aggregates. After this selection, percentage of specific cells were analysed for their specific markers.

### Cytokine,and IgA assays

Cytokines were measured in supernatant of primary cell cultures of spleen and LP after 3 days of CD3/CD28 *in vitro* re-stimulation. IFN-γ and IL-17 concentrations in supernatant were assessed by a commercial ELISA kit (Duoset R&D Systems, Lille, France) following the manufacturer’s instructions. Frozen pancreas and liver were suspended in RIPA buffer (0.5% deoxycholate, 0.1% SDS, and 1% Igepal in TBS) containing complete antiprotease cocktail (Roche, Boulogne-Billancourt, France), ground and protein concentrations in the supernatant were measured by using BCA optima kit (Interchim, Montluçon, France). TNFα, IFN-γ, IL-22 and IL-17 concentrations in pancreas and liver were assessed with a commercial ELISA kit (Duoset R&D Systems) following the manufacturer’s instructions and expressed in picograms per milligram of protein, knowing that 1 mg was loaded per well.

For faecal IgA and polymeric immunoglobulin receptor (pIgR) assays, faecal proteins were extracted mechanically in complete antiprotease cocktail (Roche) and frozen at −80 °C. IgA and pIgR concentrations were measured as described previously with sheep anti-mouse IgA (Sigma) or for coating and horseradish-peroxidase (HRP)-conjugated goat anti-mouse IgA (Sigma) for revelation^64^.

### Gene expression in gWAT

The real-time PCR was performed in a final volume of 20 µl containing 5 µl of diluted RT reaction medium, 15 µl of reaction buffer from the MESA Blue qPCR, MasterMix Plus SYBR Assay (Eurogentec, Seraing, Belgium), and the specific forward and reverse primers listed in Supplementary Table 2. For assessment of PCR efficiency, a serial dilutions curve was generated. The PCR efficiency with all primer pairs was >90%. Each assay was performed in duplicate, and the specificity of PCR reaction was assessed by evaluation of the melting curve of the PCR products. The relative expression level of the target genes was calculated by the ΔCt method (2^−ΔCt^) and were normalized to the expression of Hypoxanthine phosphoribosyltransferase (HPRT).

### Statistical analysis

Statistical analyses were performed using the GraphPad Prism 4 software (GraphPad, San Diego, CA, USA). The results were expressed as means ± s.e.m. Comparison of means was performed using nonparametric Mann-Whitney tests. The P-values of faecal microbiota results were adjusted using the Benjamini-Hochberg’s method. Values of P < 0.05 were considered significant.

## Electronic supplementary material

Supplementary Information
