# Supplementary material for: Gut dysbiosis and impairment of immune system homeostasis in perinatally-exposed mice to Bisphenol A precede obese phenotype development
Source: Sci Rep. 2017 Nov 3;7:14472. doi: 10.1038/s41598-017-15196-w (PMC5670173; doi:10.1038/s41598-017-15196-w)
Supplement: Supplementary file 1 — Supplementary Information [file 41598_2017_15196_MOESM1_ESM.pdf]

SUPPLEMENTARY INFORMATION

Gut dysbiosis and impairment of immune system homeostasis in perinatally-exposed mice to Bisphenol A precede obese phenotype development

Yann Malaisé[1], Sandrine Menard[2], Christel Cartier[1], Eric Gaultier[1], Frédéric Lasserre[3], Corinne Lencina[2], Cherryl Harkat[2], Nancy Geoffre[4], Laïla Lakhal[3], Isabelle Castan[4], Maïwenn Olier[2], Eric Houdeau[1*] & Laurence Guzylack-Piriou [1*]

[1] Intestinal Development, Xenobiotics and ImmunoToxicology team, Toxalim (Research Centre in Food Toxicology), Université de Toulouse, INRA, ENVT, INP-Purpan, UPS, Toulouse, France

[2] Neuro-Gastroenterology and Nutrition team, Toxalim (Research Centre in Food Toxicology), Université de Toulouse, INRA, ENVT, INP-Purpan, UPS, Toulouse, France

[3] Integrative Toxicology and Metabolism team, Toxalim (Research Centre in Food Toxicology), Université de Toulouse, INRA, ENVT, INP-Purpan, UPS, Toulouse, France

[4] Adipocyte secretions, obesities and related diseases team, Institut National de la Santé et de la Recherche Médicale (INSERM), Université Paul Sabatier (UPS), Unité Mixte de Recherche (UMR) 1048, Institut des Maladies Métaboliques et Cardiovasculaires (I2MC), Toulouse, France

[*] Corresponding authors:
Intestinal Development, Xenobiotics and ImmunoToxicology team, Toxalim (Research Centre in Food Toxicology), Université de Toulouse, INRA, ENVT, INP-Purpan, UPS, Toulouse, France. 180, chemin de Tournefeuille - 31027 Toulouse - FRANCE

Email: laurence.guzylack@inra.fr; eric.houdeau@inra.fr

Supplementary Table 1. List of isotype controls used for surface and intracellular antigen staining, including provider and references.

Antigen	Provider	Isotype control	References
CD4	BD Biosciences	PE rat IgG2a, κ isotype control	553989
CD25	BD Biosciences	PE-Cy7™ rat IgG1, λ isotype control	552869
CD3	BD Biosciences	FITC hamster IgG1, κ isotype control	553971
IFN-g	BD Biosciences	Alexa fluor 647 rat IgG1, κ isotype control	557731
T-bet	BD Biosciences	PE mouse IgG1, κ isotype control	556320
IL-17	BD Biosciences	Alexa fluor 647 rat IgG1, κ isotype control	557731
ROR-gt	BD Biosciences	Alexa fluor 647 rat IgG2a, κ isotype control	557715
CD11b	BD Biosciences	APC-Cy™7 rat IgG2b, κ isotype control	552773
CD45.2	BD Biosciences	PerCP-Cy™5.5 mouse IgG2a, κ isotype control	550927
CD11c	BD Biosciences	PE hamster IgG1, λ1 isotype control	553954
CD301	eBioscience	efluor ® 660 rat IgG2a κ isotype control	50-4321
F4/80	eBioscience	PE-cy7 rat IgG2a κ isotype control	25-432
FoxP3	eBioscience	efluor ® 660 rat IgG2a κ isotype control	50-4321

Supplementary Table 2. Forward and reverse primer sequences used for real time quantitative RT-PCR analysis.

Gene		Sequence
IFN-γ	Forward	GGAGGAACTGGCAAAAGGATGG
	Reverse	TGTTGCTGATGGCCTGATTGTC
IL-17	Forward	CAGCAGCGATCATCCCTCAA
	Reverse	TTTCCCTCCGCATTGACACA
IL-1β	Forward	CAACCAACAAGTGATATTCTCGATG
	Reverse	GATCCACACTCTCCAGCTGCA
IL-6	Forward	GCCCACCAAGAACGATAGTCA
	Reverse	CAAGAAGGCAACTGGATGGAA
Inos	Forward	CACCTTGGACTTCACCCAGT
	Reverse	ACCACTCGTACTTGGGATGT
TNF-α	Forward	TGGGACAGTGACCTGGACTGT
	Reverse	TTCGGAAAGCCCATTTGAGT
HPRT	Forward	TGGCCATCTGCCTAGTAAAGC
	Reverse	GGACGCAGCAACTGACATTTC

IFN-γ, Interferon-gamma; IL-17, Interleukin-17 ; IL-1β, Interleukin-1β ; IL-6, Interleukin-6 ; Inos, inducible Nitric oxide synthases ; TNF-α, Tumor necrosis factor-α ; HPRT, Hypoxanthine phosphoribosyltransferase

Supplementary Figure S1. Isotype controls for M1/M2 characterization by flow cytometry.

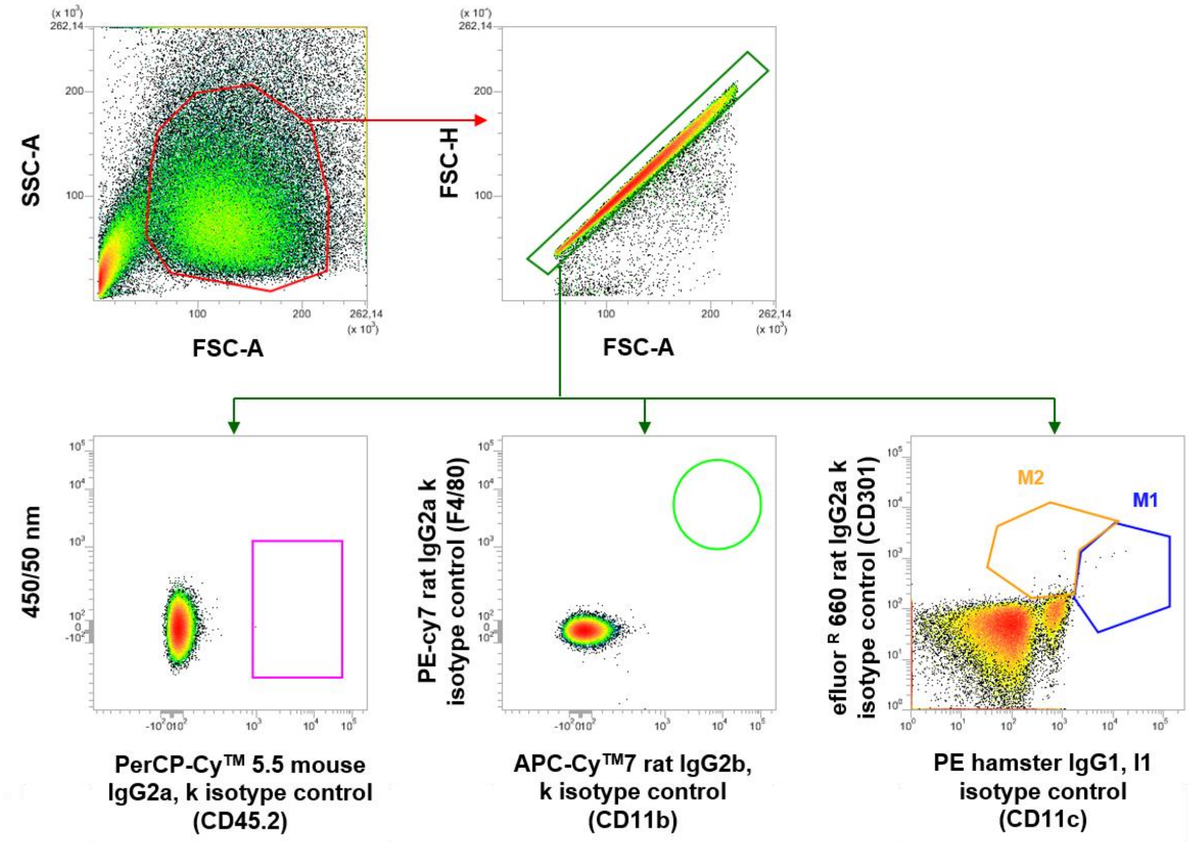

Isotype controls of Figure 3a and b.

Supplementary Figure S2. FSS and HRP permeability of jejunum from PND45 and PND170 male offspring mice.

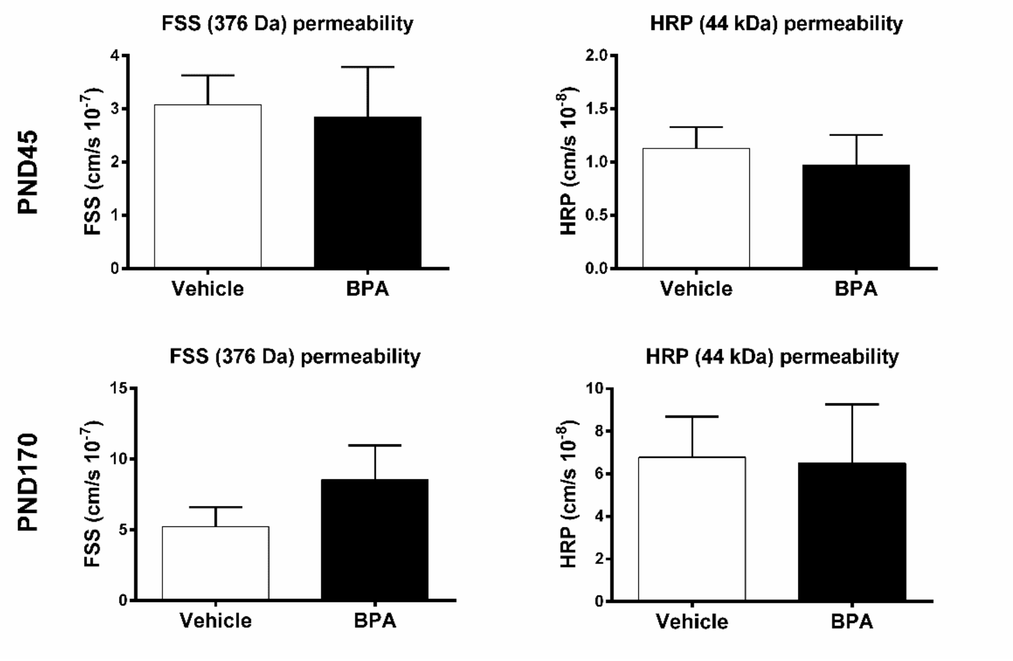

Jejunal fragments from male offspring at PND45 and PND170 were mounted in Ussing chambers (Easy Mount, Physiologic Instruments) exposing a surface area measuring 0.1 cm^2. They were bathed on each side with 1 ml of oxygenated thermostated Kreb's solution (Sigma-Aldrich, Saint-Quentin Fallavier, France). Electrical parameters, including potential difference, short-circuit current (Isc) and total electrical resistance (R), were recorded at regular intervals during the 2-hour period of experimentation. Horseradish peroxidase (HRP 44kDa) (Sigma) transport was measured as an index of macromolecular permeability, and FSS (376Da) (Sigma) epithelial passage was measured as a marker of paracellular permeability to small molecules. After equilibration of electrical parameters, HRP was added to the mucosal compartment at a final concentration of 0.4 mg/ml, and FSS at a final concentration of 40g/ml. The two markers were applied simultaneously in the mucosal compartment. Epithelial permeability to total HRP was determined by an ELISA. Briefly, 96-wells flat-bottomed black plates (Greiner, Thermo Scientific, Dutcher, Brumath, France) were coated overnight at room temperature with 50µl of 10µg/ml mouse polyclonal to HRP (Abcam, Paris, France) in PBS. Plates were blocked with PBS-5% fetal bovine serum (FBS) before incubation with serosal compartments of Ussing chamber. Rabbit polyclonal anti HRP biotin (Abcam) was added at a concentration of 1.6 µg/ml before revelation with SPRD (SpectralRed conjugate) conjugated streptavidin (Southern Biotech) was added for 20 minutes and fluorescence intensity measured 565nm/666nm using an automatic Infinite M200 microplate reader (Tecan, Lyon, France). Epithelial permeability to FSS was determined by measuring the fluorescence intensity 485nm/525nm using an automatic Infinite M200 microplate reader (Tecan). Permeability was calculated as the ratio of flux/concentration, and expressed as cm/second.
